# High-quality permanent draft genome sequence of the *Lebeckia ambigua*-nodulating *Burkholderia* sp. strain WSM4176

**DOI:** 10.1186/s40793-015-0072-3

**Published:** 2015-10-16

**Authors:** Sofie E. De Meyer, Rui Tian, Rekha Seshadri, TBK Reddy, Victor Markowitz, Natalia Ivanova, Amrita Pati, Tanja Woyke, Nikos Kyrpides, Ron Yates, John Howieson, Wayne Reeve

**Affiliations:** Centre for Rhizobium Studies, Murdoch University, Murdoch, WA Australia; DOE Joint Genome Institute, Walnut Creek, CA USA; Biological Data Management and Technology Center, Lawrence Berkeley National Laboratory, Berkeley, CA USA; Department of Biological Sciences, King Abdulaziz University, Jeddah, Saudi Arabia; Department of Agriculture and Food, Western Australia, Australia

**Keywords:** Root-nodule bacteria, Nitrogen fixation, Rhizobia, *Betaproteobacteria*, GEBA-RNB

## Abstract

*Burkholderia* sp. strain WSM4176 is an aerobic, motile, Gram-negative, non-spore-forming rod that was isolated from an effective N_2_-fixing root nodule of *Lebeckia ambigua* collected in Nieuwoudtville, Western Cape of South Africa, in October 2007. This plant persists in infertile, acidic and deep sandy soils, and is therefore an ideal candidate for a perennial based agriculture system in Western Australia. Here we describe the features of *Burkholderia* sp. strain WSM4176, which represents a potential inoculant quality strain for *L. ambigua,* together with sequence and annotation. The 9,065,247 bp high-quality-draft genome is arranged in 13 scaffolds of 65 contigs, contains 8369 protein-coding genes and 128 RNA-only encoding genes, and is part of the GEBA-RNB project proposal (Project ID 882).

## Introduction

Leguminous pasture species are important in Western Australian agriculture because the soils are inherently infertile. Together with changing patterns of rainfall, this agricultural system cannot continue to rely on the current commercially used annual legumes. Deep-rooted herbaceous perennial legumes including *Rhynchosia* and *Lebeckia* species from the Cape Floristic Region in South Africa have been investigated because of their adaptation to acid and infertile soils [1–3]. These plants naturally occur in the CFR, which is one of the richest areas for plants in the world and covers 553,000 ha of land protected by the UNESCO as important world heritage. Elevations in this area range from 2077 m in the Groot Winterhoek to sea level in the De Hoop Nature Reserve. Moreover, a great part of the area is characterized by mountains, rivers, waterfalls and pools. In areas where *Lebeckia ambigua* is native, rainfall ranges between 150 and 400 mm annually. Parts of the CFR have thus similar soil and climate conditions to Western Australia.

In four expeditions to the Western Cape of South Africa, held between 2002 and 2007, nodules and seeds were collected and stored as previously described [4]. The isolation of bacteria from these nodules gave rise to a collection of 23 strains that were identified as *Burkholderia*. Unlike most of the previously studied rhizobial *Burkholderia* strains, this South African group appears to associate with papilionoid forage legumes, rather than *Mimosa* species. WSM4176 belongs to a subgroup of strains that were isolated in 2004 from *Lebeckia ambigua* nodules collected near Nieuwoudtville in the Western Cape of South Africa [3]. The site of collection was moderately grazed rangeland field owned by the Louw family, and the soil was composed of stony-sand with a pH of 6. *Burkholderia* sp. strain WSM4176 is highly effective at fixing nitrogen with *Lebeckia ambigua*, with which it forms crotaloid, indeterminate, nodules [3].

WSM4176 represents thus a potential inoculant quality strain for *Lebeckia ambigua*, which is being developed as a grazing legume adapted to infertile soils that receive 250–400 mm annual rainfall, where climate change has necessitated the domestication of agricultural species with altered characteristics. Therefore, this strain is of special interest to the IMG/GEBA project. Here we present a summary classification and a set of general features for *Burkholderia* sp. strain WSM4176 together with the description of the complete genome sequence and annotation.

## Organism information

### Classification and features

**Fig. 1:**
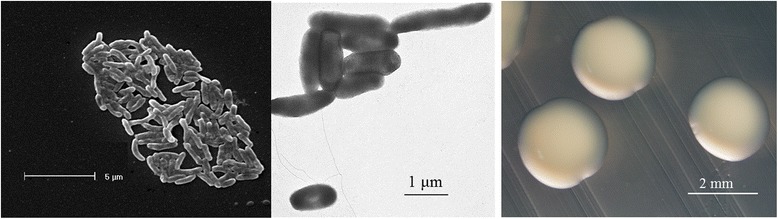
Images of *Burkholderia* sp. strain WSM4176 using scanning (*Left*) and transmission (*Center*) electron microscopy and the appearance of colony morphology on solid media (*Right*)

*Burkholderia* sp. strain WSM4176 is a motile, Gram-negative, non-spore-forming rod (Fig. 1 Left, Center) in the order *Burkholderiales* of the class *Betaproteobacteria*. The rod-shaped form varies in size with dimensions of 0.1–0.2 μm in width and 2.0–3.0 μm in length (Fig. 1 Left). It is fast growing, forming 0.5–1 mm diameter colonies after 24 h when grown on half Lupin Agar [5] and TY [6] at 28 °C. Colonies on ½LA are white-opaque, slightly domed, moderately mucoid with smooth margins (Fig. 1 Right).

**Fig. 2:**
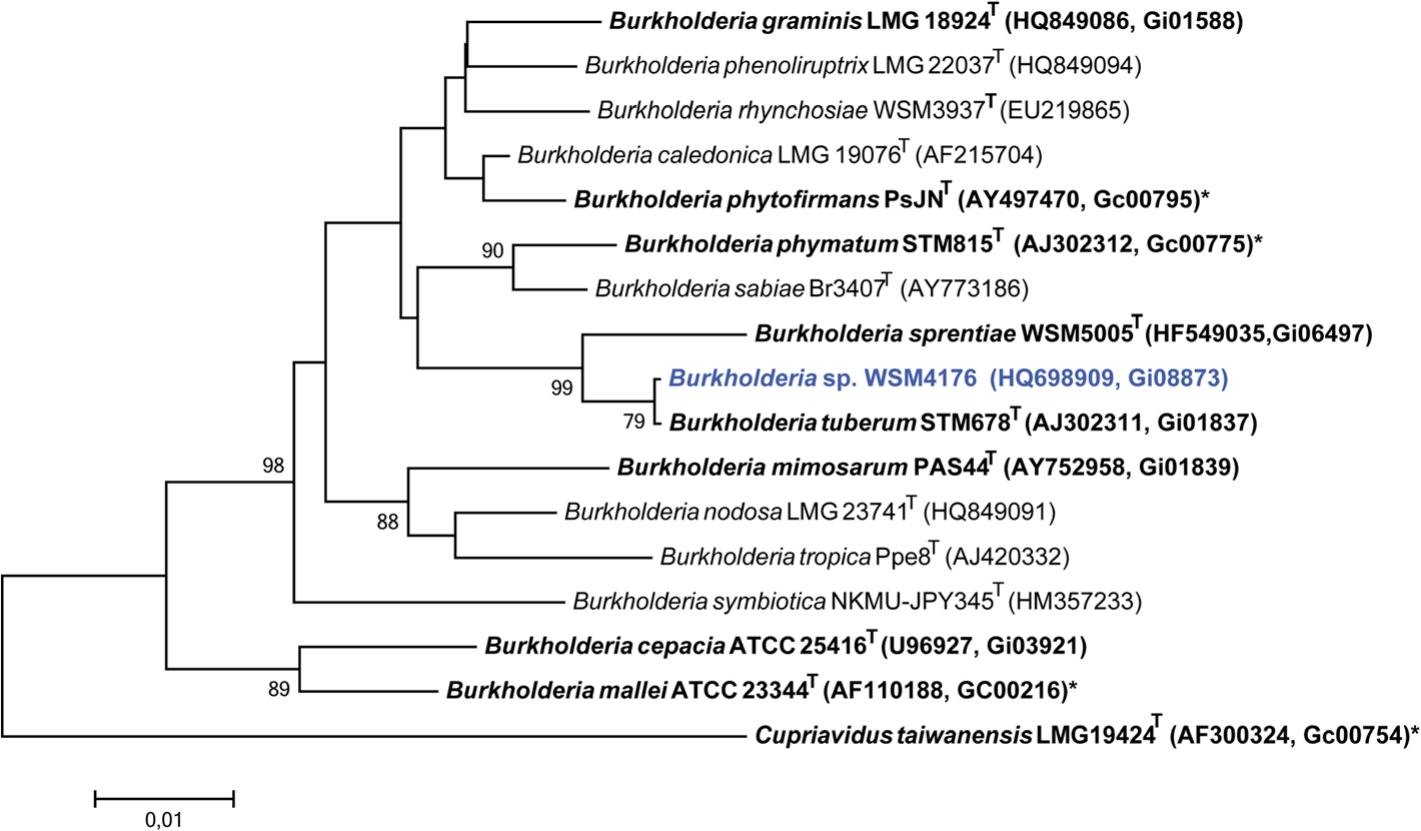
Phylogenetic tree highlighting the position of *Burkholderia* sp. strain WSM4176 (shown in blue print) relative to other type and non-type strains in the *Burkholderia* genus (1322 bp internal region). *Cupriavidus taiwanensis* LMG 19424^T^ was used as outgroup. All sites were informative and there were no gap-containing sites. Phylogenetic analyses were performed using MEGA, version 5.05 [27]. The tree was build using the maximum likelihood method with the General Time Reversible model. Bootstrap analysis with 500 replicates was performed to assess the support of the clusters. Type strains are indicated with a superscript T. Strains with a genome sequencing project registered in GOLD [9] are in bold print and the GOLD ID is mentioned after the NCBI accession number. Published genomes are designated with an asterisk

Figure 2 shows the phylogenetic relationship of *Burkholderia* sp. strain WSM4176 in a 16S rRNA gene sequence based tree. This strain clusters closest to *Burkholderia tuberum* STM678^T^ and *Burkholderia phenoliruptrix* AC1100^T^ with 99.86 and 97.28 % sequence identity, respectively. Minimum Information about the Genome Sequence is provided in Table 1.

**Table 1:** Classification and general features of *Burkholderia* sp. strain WSM4176 in accordance with the MIGS recommendations [28] published by the Genome Standards Consortium [29]

MIGS ID	Property	Term	Evidence code
	Classification	Domain *Bacteria*	TAS [30]
		Phylum *Proteobacteria*	TAS [31, 32]
		Class *Betaproteobacteria*	TAS [33]
		Order *Burkholderiales*	TAS [34]
		Family *Burkholderiaceae*	TAS [35]
		Genus *Burkholderia*	TAS [36]
		Species *Burkholderia* sp.	TAS [3]
		(Type) strain WSM4176	TAS [3]
	Gram stain	Negative	IDA [36]
	Cell shape	Rod	IDA
	Motility	Motile	IDA
	Sporulation	Non-sporulating	IDA [36]
	Temperature range	Not reported	
	Optimum temperature	28 °C	IDA
	pH range; Optimum	Not reported	
	Carbon source	Not reported	
MIGS-6	Habitat	Soil, root nodule on host	TAS [3]
MIGS-6.3	Salinity	Not reported	
MIGS-22	Oxygen requirement	Aerobic	IDA
MIGS-15	Biotic relationship	Free living, symbiotic	TAS [3]
MIGS-14	Pathogenicity	Non-pathogenic	NAS
MIGS-4	Geographic location	South Africa	TAS [3]
MIGS-5	Sample collection	2007	TAS [3]
MIGS-4.1	Latitude	−31.381	TAS [3]
MIGS-4.2	Longitude	19.30	TAS [3]
MIGS-4.4	Altitude	789 m	IDA

Evidence codes – *IDA* inferred from direct assay, *TAS* traceable author statement (i.e., a direct report exists in the literature), *NAS* non-traceable author statement (i.e., not directly observed for the living, isolated sample, but based on a generally accepted property for the species, or anecdotal evidence). These evidence codes are from the Gene Ontology project [37]

### Symbiotaxonomy

*Burkholderia* sp. strain WSM4176 belongs to a group of *Burkholderia* strains that nodulate papilionoid forage legumes rather than the classical *Burkholderia* hosts *Mimosa* spp. (Mimosoideae) [7]. *Burkholderia* sp. strain WSM4176 was assessed for nodulation and nitrogen fixation on three separate *L. ambigua* genotypes (CRSLAM-37, CRSLAM-39 and CRSLAM-41) [3]. Strain WSM4176 could nodulate and fix effectively on CRSLAM-39 and CRSLAM-41 but was partially effective on CRSLAM-37 [3].

## Genome sequencing information

### Genome project history

This organism was selected for sequencing on the basis of its environmental and agricultural relevance to issues in global carbon cycling, alternative energy production, and biogeochemical importance, and is part of the Genomic Encyclopedia of Bacteria and Archaea, The Root Nodulating Bacteria chapter project at the U.S. Department of Energy, Joint Genome Institute for projects of relevance to agency missions [8]. The genome project is deposited in the Genomes OnLine Database [9] and the high-quality permanent draft genome sequence in IMG [10]. Sequencing, finishing and annotation were performed by the JGI using state of the art sequencing technology [11]. A summary of the project information is shown in Table 2.

**Table 2:** Genome sequencing project information for *Burkholderia* sp. strain WSM4176

MIGS ID	Property	Term
MIGS-31	Finishing quality	High-quality-permanent-draft
MIGS-28	Libraries used	Illumina CLIP PE and Illumina Std PE Unamplified
MIGS-29	Sequencing platforms	Illumina HiSeq 2000
MIGS-31.2	Fold coverage	361 × Illumina
MIGS-30	Assemblers	ALLPATHS V.r41554
MIGS-32	Gene calling methods	Prodigal 1.4, GenePRIMP
	Locus Tag	B014
	Genbank ID	ARCY00000000
	Genbank Date of Release	July 11, 2014
	GOLD ID	Gi08873
	BIOPROJECT	PRJNA169686
MIGS-13	Source Material Identifier	WSM4176
	Project relevance	Symbiotic N_2_fixation, agriculture

### Growth conditions and genomic DNA preparation

*Burkholderia* sp. strain WSM4176 was grown to mid logarithmic phase in TY rich media [6] on a gyratory shaker at 28 °C. DNA was isolated from 60 mL of cells using a CTAB bacterial genomic DNA isolation method [12].

### Genome sequencing and assembly

The genome of *Burkholderia* sp. strain WSM4176 was sequenced at the DOE Joint Genome Institute (JGI) using Illumina data [13]. For this genome, we constructed and sequenced an Illumina short-insert paired-end library with an average insert size of 270 bp which generated 7,496,994 reads and an Illumina long-insert paired-end library with an average insert size of 6899.89 +/− 882.09 bp which generated 11,773,350 reads totaling 2891 Mbp of Illumina data (unpublished, Feng Chen). All general aspects of library construction and sequencing performed at the JGI can be found at the JGI’s web site [11]. The initial draft assembly contained 66 contigs in eight scaffold(s). The initial draft data was assembled with Allpaths, version r41554 [14], and the consensus was computationally shredded into 10 Kbp overlapping fake reads (shreds). The Illumina draft data was also assembled with Velvet, version 1.1.05 [15], and the consensus sequences were computationally shredded into 1.5 Kbp overlapping fake reads (shreds). The Illumina draft data was assembled again with Velvet using the shreds from the first Velvet assembly to guide the next assembly. The consensus from the second Velvet assembly was shredded into 1.5 Kbp overlapping fake reads. The fake reads from the Allpaths assembly and both Velvet assemblies and a subset of the Illumina CLIP paired-end reads were assembled using parallel phrap, version 4.24 (High Performance Software, LLC). Possible mis-assemblies were corrected with manual editing in Consed [16–18]. Gap closure was accomplished using repeat resolution software (Wei Gu, unpublished), and sequencing of bridging PCR fragments with Sanger and/or PacBio (unpublished, Cliff Han) technologies. For improved high quality draft and non-contiguous finished projects, one round of manual/wet lab finishing may have been completed. Primer walks, shatter libraries, and/or subsequent PCR reads may also be included for a finished project. A total of 11 PCR PacBio consensus sequences were completed to close gaps and to raise the quality of the final sequence. The total size of the genome is 9.1 Mb and the final assembly is based on 2891 Mbp of Illumina draft data, which provides an average 318× coverage of the genome.

### Genome annotation

Genes were identified using Prodigal [19] as part of the DOE-JGI Annotation pipeline [17], followed by a round of manual curation using the JGI GenePRIMP pipeline [20]. The predicted CDSs were translated and used to search the National Center for Biotechnology Information nonredundant database, UniProt, TIGRFam, Pfam, PRIAM, KEGG, COG, and InterPro databases. These data sources were combined to assert a product description for each predicted protein. Non-coding genes and miscellaneous features were predicted using tRNAscan-SE [21], RNAMMer [22], Rfam [23], TMHMM [24] and SignalP [23]. Additional gene prediction analyses and functional annotation were performed within the Integrated Microbial Genomes platform [24].

### Genome properties

**Fig. 3:**
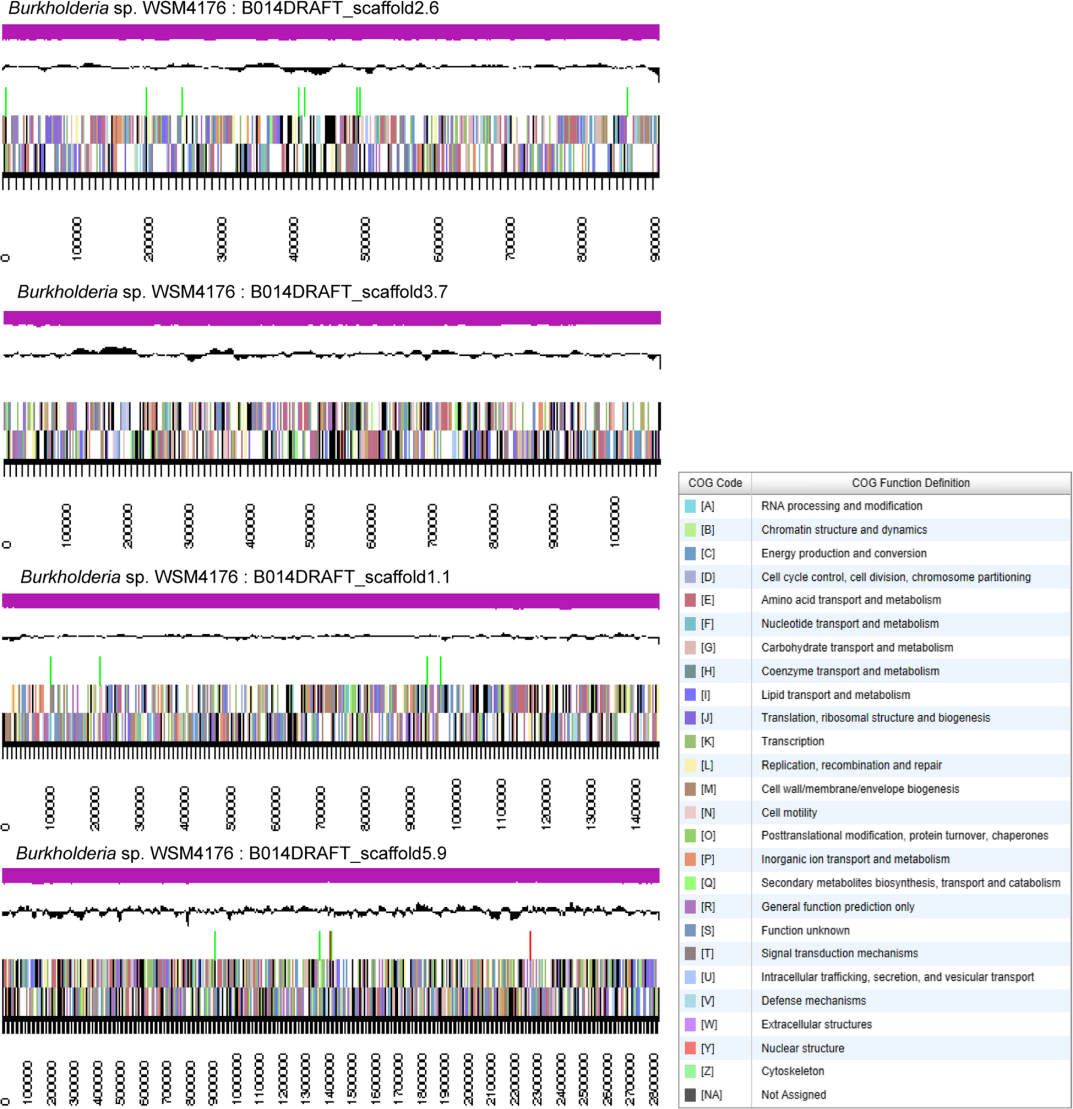
Graphical map of the genome of *Burkholderia* sp. strain WSM4176. First four large scaffolds are shown according to size. From the bottom to the top of each scaffold: Genes on forward strand (color by COG categories as denoted by the IMG platform), Genes on reverse strand (color by COG categories), RNA genes (tRNAs green, sRNAs red, other RNAs black), GC content, GC skew

The genome is 9,065,247 nucleotides with 62.89 % GC content (Table 3) and comprised of 13 scaffolds and 65 contigs (Fig. 3). From a total of 8497 genes, 8369 were protein encoding and 128 RNA only encoding genes. The majority of genes (75.46 %) were assigned a putative function whilst the remaining genes were annotated as hypothetical. The distribution of genes into COGs functional categories is presented in Table 4.

**Table 3:** Genome statistics for *Burkholderia* sp. strain WSM4176

Attribute	Value	% of total
Genome size (bp)	9,065,247	100.00
DNA coding (bp)	7,632,174	84.19
DNA G+C (bp)	5,701,432	62.89
DNA scaffolds	13	
Total genes	8497	100.00
Protein-coding genes	8369	98.49
RNA genes	128	1.51
Pseudo genes	0	0.00
Genes in internal clusters	648	7.63
Genes with function prediction	6412	75.46
Genes assigned to COGs	5491	64.62
Genes with Pfam domains	6766	79.63
Genes with signal peptides	738	8.69
Genes with transmembrane helices	1865	21.95
CRISPR repeats	0	0.00

**Table 4:** Number of protein coding genes of *Burkholderia* sp. strain WSM4176 associated with the general COG functional categories

Code	Value	% age	COG category
J	200	3.21	Translation
A	1	0.02	RNA processing and modification
K	596	9.55	Transcription
L	299	4.79	Replication, recombination and repair
B	1	0.02	Chromatin structure and dynamics
D	38	0.61	Cell cycle control, mitosis and meiosis
V	74	1.19	Defense mechanisms
T	270	4.33	Signal transduction mechanisms
M	389	6.23	Cell wall/membrane biogenesis
N	105	1.68	Cell motility
U	146	2.34	Intracellular trafficking and secretion
O	172	2.76	Posttranslational modification, protein turnover, chaperones
C	461	7.39	Energy production conversion
G	495	7.93	Carbohydrate transport and metabolism
E	611	9.79	Amino acid transport metabolism
F	101	1.62	Nucleotide transport and metabolism
H	210	3.37	Coenzyme transport and metabolism
I	323	5.18	Lipid transport and metabolism
P	317	5.08	Inorganic ion transport and metabolism
Q	225	3.61	Secondary metabolite biosynthesis, transport and catabolism
R	727	11.65	General function prediction only
S	479	7.68	Function unknown
-	3006	35.38	Not in COGS

The total is based on the total number of protein coding genes in the genome

## Conclusion

*Burkholderia* sp. WSM4176 belongs to a group of Beta-rhizobia isolated from *Lebeckia ambigua* from the fynbos biome in South Africa [3]. WSM4176 is phylogeneticaly most closely related to *Burkholderia tuberum* STM678^T^. Both STM678^T^ and WSM4176 have comparable genome sizes, 8.3–9.1 respectively. Recently, two more genomes from strains originating from *Lebeckia ambigua* were investigated, *Burkholderia dilworthii*WSM3556^T^ and *Burkholderia sprentiae*WSM5005^T^ [25]. Both of these strains have a genome size of 7.7 Mbp, which is considerably smaller than WSM4176. All four strains, STM678^T^, WSM3556^T^, WSM4176 and WSM5005^T^, contain a large number of genes assigned to transport and metabolism of amino acids (9.79–10.94 %) and carbohydrates (7.93–8.38 %), and transcription (9.55–9.94 %). Interestingly, STM678^T^ was initially isolated from *Aspalathus* species but does not nodulate this host, however it has been shown to nodulate *Cyclopia* species from the same fynbos biome in South Africa as *Lebeckia ambigua* [26]. Considering the ability of these strains to nodulate and fix nitrogen effectively with legumes, they share in common many of the genes responsible for the nitrogenase pathway (IMG pathway number 798). The genome sequence of WSM4176 provides thus an unprecedented opportunity to study the host range and nitrogen fixation capacities of these fynbos bacteria.

## Abbreviations

GEBA-RNB: Genomic encyclopedia of bacteria and archaea – root nodule bacteria
JGI: Joint genome institute
TY: Trypton yeast
CTAB: Cetyl trimethyl ammonium bromide
WSM: Western Australian soil microbiology
BNF: Biological nitrogen fixation
CFR: Cape floristic region
